# A Decade of Progress in Gene Targeted Therapeutic Strategies in Duchenne Muscular Dystrophy: A Systematic Review

**DOI:** 10.3389/fbioe.2022.833833

**Published:** 2022-03-23

**Authors:** Lam Chung Liang, Nadiah Sulaiman, Muhammad Dain Yazid

**Affiliations:** Centre for Tissue Engineering and Regenerative Medicine, Faculty of Medicine, Universiti Kebangsaan Malaysia Medical Centre, Kuala Lumpur, Malaysia

**Keywords:** Duchenne muscular dystrophy, DMD, gene therapy, CRISPR/Cas9, exon skipping

## Abstract

As one of the most severe forms of muscle dystrophy, Duchenne muscular dystrophy (DMD) results in progressive muscle wasting, ultimately resulting in premature death due to cardiomyopathy. In the many years of research, the solution to DMD remains palliative. Although numerous studies including clinical trials have provided promising results, approved drugs, even, the therapeutic window is still minimal with many shortcomings to be addressed. Logically, to combat DMD that arose from a single genetic mutation with gene therapy made sense. However, gene-based strategies as a treatment option are no stranger to drawbacks and limitations such as the size of the dystrophin gene and possibilities of vectors to elicit immune responses. In this systematic review, we aim to provide a comprehensive compilation on gene-based therapeutic strategies and critically evaluate the approaches relative to its efficacy and feasibility while addressing their current limitations. With the keywords “DMD AND Gene OR Genetic AND Therapy OR Treatment,” we reviewed papers published in Science Direct, PubMed, and ProQuest over the past decade (2012–2021).

## Graphical Abstract

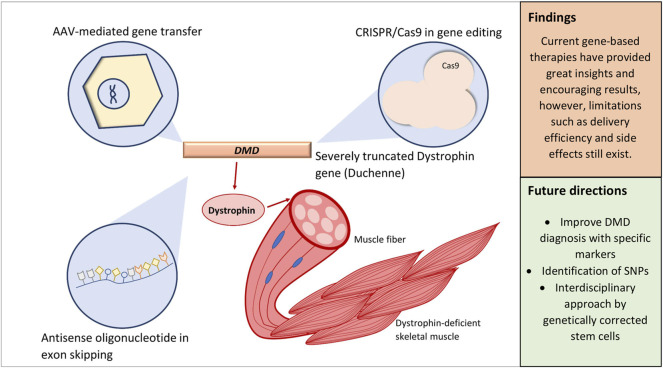

## Introduction

The DMD gene in its entirety covers 2.4 Mb in size, making it the largest known human gene with 79 exons (Gao and McNally, 2015; Kumar et al., 2020). At such size, this gene provides the instruction to give rise to a 427 kD protein, dystrophin, that is integral to sarcolemmal integrity (Hoffman, 2020). Such size also meant that spontaneous mutations occur at a high rate, which is evidenced by *de novo* mutations reported in one-third of cases (Juan-Mateu et al., 2015). In general, large mutations involving deletion or duplication of one of more exons account for an approximate of 60–70%, while the remaining 25–35% are small mutations comprised of missense, nonsense, and frame-shift mutations (Kong et al., 2019). The mutational spectrum of DMD exhaustively varies; however, it was discovered that mutational hotspots exist along the gene, notably duplication clusters in exons 2–10 and deletion clusters in exons 43–55 (Aartsma-Rus et al., 2016; Niks and Aartsma-Rus, 2017; Min et al., 2020). In rare cases, deep intronic copy number variations (CNVs) and mid-intronic mutations that produce cryptic exons or pseudoexons may potentially be deleterious in nature disrupting the normal reading frame of the DMD gene (Khelifi et al., 2011; Trabelsi et al., 2014; Greer et al., 2015; Keegan et al., 2019).

While it is known that DMD is an X-linked recessive disorder that predominantly affects males whereas females largely remain as asymptomatic carriers, the phenotypes of DMD are at times expressed in female carriers manifesting (2.5–10%) muscle weakness and cramps (Lee et al., 2015; Zhong et al., 2019). This can be associated with a skewed inactivation of the X chromosome carrying the normal gene, or in the case of chromosomal aberrations in carriers with Turner syndrome (Giliberto et al., 2014; Lee et al., 2015). Interestingly, the aforementioned association was challenged by a study conducted by Brioschi et al. (2012), in which it was reported that there was a lack of relationship between X-chromosome inactivation and dystrophinopathic phenotype observed in female carriers. The findings, therefore, suggest that the dystrophin level may be the key behind the expression of the DMD phenotype.

This brings us into emphasizing the role of dystrophin and its expression, which, irrespective of gender, has an impact on the phenotype and progression of the disease. The loss of functional dystrophin expression has a direct effect on the dystrophin-associated glycoprotein complex (DGC) resulting in membrane instability and increased susceptibility to injury that will eventually be replaced by fibroadipose tissues (Falzarano et al., 2015; Niks and Aartsma-Rus, 2017). That said, therapeutic strategies focusing on correcting and restoring dystrophin expression have been in development rigorously.

When Rosenberg et al. (1990) first demonstrated a retroviral-mediated gene transduction in modifying tumor-infiltrating lymphocytes (TILs), it was both a breakthrough and a landmark. The study had proven two key events: 1) It is possible to genetically modify human cells, and 2) the approach was safe and feasible that no adverse effects were observed upon the introduction of the modified cell. Cumulatively as of 2017, there are almost 2,600 clinical trials employing gene-based strategies, which are either approved, completed, or still ongoing (Ginn et al., 2018). While success stories along the years were not without setbacks, limitations, and public prejudice, numerous studies emerging in the third decade have achieved in-depth understanding, demonstrating feasibility of gene therapy and at the same time pinpointing current limitations to be revisited and improved.

The trend in current DMD research can be distinguished based on distinct niches: gene therapy, which is of our interest, pharmacological therapy (Guglieri et al., 2017; Bhattacharya et al., 2018), cell therapy (Dai et al., 2018; Siemionow et al., 2018; Jelinkova et al., 2019), improved disease management (Sheehan et al., 2018; Adorisio et al., 2020), and therapy on the downstream pathology of DMD (Guiraud and Davies, 2017). Efforts to combat DMD through gene-based intervention are of exceptional interest as the disease itself is due to single-gene mutations. The idea of correcting specific mutations also draws in opportunities for a personalized approach; for example, the correction of exon 51 via exon skipping is applicable to about 14% of patients (Lim et al., 2017; Dzierlega and Yokota, 2020). These strategies include, but are not limited to, employing the CRISPR/Cas9 system, adeno-associated virus (AAV) vectors, and antisense oligonucleotides (AOs) of various constructs (Łoboda and Dulak, 2020).

In justifying the focus toward gene therapy, this approach allows defective genes to be corrected at an earlier stage (Wolf et al., 2019; Palanski et al., 2020). In contrast, the emerging stem cell-based therapy in DMD, which also predominantly exists in laboratories, is impeded by poor systemic delivery efficacy and reliance of immunosuppressants over graft rejection (Biressi et al., 2020). While sharing almost the same set of challenges, the plasticity and flexibility of gene-based therapies over the maturation of this field have proven its ability to alternatively overcome some of these challenges, providing promise in treating diseases that were once incurable (Dunbar et al., 2018). Although the trajectory of DMD research with regard to gene therapy has dramatically progressed in recent years, realistic therapeutic efficacy is still minimal considering that challenges and limitations still exist. Therefore, our objective in this systematic review is to provide critical assessment of current gene-based therapeutic strategies while addressing their limitations. Through systematic reviews, relevant data could be reliably curated and analyzed using scientific strategies such as risk of bias analysis and guidelines set. Ultimately, this avoids the reporting of biased results and retains the presentation of high-quality results (Ahn and Kang, 2018; Henry et al., 2018).

## Materials and Methods

### Search Strategy

In this systematic review, we assemble and report in accordance with the guidelines set in Preferred Reporting Items for Systematic Reviews and Meta-analyses (PRISMA) (Supplementary Table S1). For the retrieval of articles, a comprehensive systematic screening through three online databases, Science Direct, PubMed, and ProQuest, was performed in accordance with PRISMA guidelines (Page et al., 2021). Original research articles relevant to gene-based therapeutic strategies on DMD were searched employing the following keywords: DMD AND Gene OR Genetic AND Therapy OR Treatment.

### Inclusion and Exclusion Criteria

**FIGURE 1:**
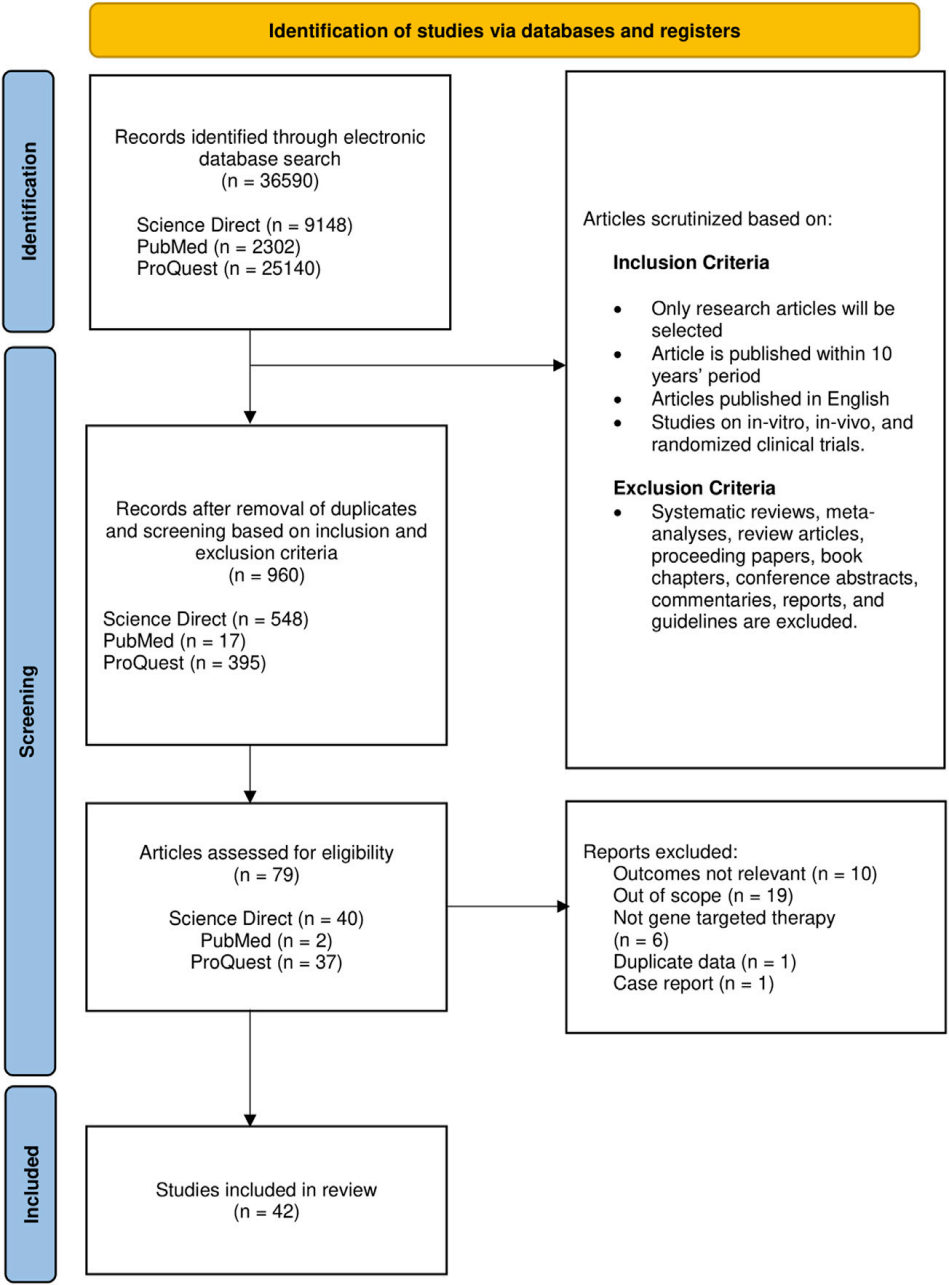
Flow chart representing article selection process based on PRISMA.

Full-text articles in English with publication period set from 2012 to 2021 were included. Articles obtained were exclusively original research articles including *in vitro*, *in vivo*, and clinical trials. While adhering to the exclusion criteria (Figure 1), the reference lists in systematic reviews were screened to identify potential studies prior to exclusion. Articles relevant to the gene-based therapeutic strategies irrespective of the type of intervention were selected to be evaluated thoroughly and independently by three reviewers.

### Data Extraction, Quality Evaluation, and Risk-Bias Assessment

Similarly, data were extracted from each eligible article by three reviewers working independently. The selected articles were screened to meet specific selection criteria. Articles with outcomes that were not relevant or with studies that were out of our scope of interest were excluded. The extracted data are tabulated concisely in the following order: 1) References, 2) Aim, 3) Tested Compound/Treatment Strategy, 4) Method of Intervention, 5) Additional Information, 6) Findings, and 7) Conclusion and Impact on DMD Therapy. The selected studies were validated with a modified version of Office of Health Assessment and Translation (OHAT). The study quality relative to risk of bias was classified as definitely low risk, probably low risk, probably high risk, definitely high risk, and not applicable concerning the following fields: reporting bias, performance bias, detection bias, and selection bias.

## Results and Discussion

### Search Result and Study Characteristics

The initial search has identified 36,590 articles, to which only 960 remain after removal based on the criteria mentioned; duplicates were also removed in this stage. After initial screening for eligibility, 79 (8.2%) articles were retained for further evaluation of the title and abstract on the basis of relevance. Of these 79, 42 (53.2%) met all the eligibility criteria set and hence were included (Figure 1 and Supplementary Table S2). Pertaining to our selection strategy based on relevance, only studies centered on gene therapy on DMD were selected, while articles with only brief mentions of the aforementioned focus, genetic interventions on other types of muscular dystrophies, and studies on cellular signaling events in DMD were deemed as articles with outcomes not relevant.

In summary, there are 23 studies on exon skipping, 8 on gene editing, 5 on gene transfer as means of intervention, 5 studies that used microdystrophin gene therapy, and 1 on RNA interference (Supplementary Table S3). It is, however, necessary to underline that the frequency of studies does not indicate superiority over any of their counterparts. Most of the studies were performed *in vivo* (*n* = 22; 52.4%), while 13 (31%) were performed in combination of *in vitro* and *in vivo*. In one of the studies, *in silico* analysis was performed in order to predict individual exon skipping efficiencies prior to selecting the AOs for *in vitro* and *in vivo* studies (Echigoya et al., 2017).

### Quality Evaluation

Upon evaluation, all of the studies show low risk of bias. However, it is noteworthy that although the study by Hayashita-Kinoh et al. (2015) did not perform randomization of microdystrophin gene administration on the canine models, it was ruled to be of low bias as the parameters and the result reported were all quantitative. Out of 42 studies, 9 were evaluated to carry the possibility of a low-risk bias (+), which were all due to indirect and unclear mention of statistical tests performed, therefore also slightly affecting the confidence of the outcome assessment of 4 studies (Supplementary Table S4).

### Exon Skipping

DMD pathology is directly associated with loss of dystrophin expression due to mutations of the normal reading frame. Restoration of the disrupted reading frame can be achieved through exon skipping that utilizes AOs, short single-stranded deoxynucleotides that induce endonuclease-mediated knockdown in the DMD transcript by targeting dystrophin pre-mRNA and inhibit mRNA translation. Consequently, this mechanism converts the severe out-of-frame mutation into an in-frame mutation (Crooke, 2017; Rinaldi and Wood, 2018). In this approach, functional dystrophin, though still internally truncated, can be produced, essentially converting the DMD phenotype to the milder Becker muscular dystrophy (Findlay et al., 2015). Given such specificity, exon skipping can be personalized to benefit larger groups of DMD patients. Eteplirsen, which is an AO that was granted accelerated approval by the FDA in 2016, induces exon 51 skipping that could benefit around 14% of DMD patients (Syed, 2016; Łoboda and Dulak, 2020). However, the efficacy of Eteplirsen is rather controversial as claims of Eteplirsen demonstrating significant dystrophin restoration in phase III of the PROMOVI trial (NCT02255552) were only evidenced by 12 evaluable patients (Syed, 2016; Akpulat et al., 2018). The relatively short half-life of AOs and its rapid clearance from the circulation were two challenges that were continuously addressed; the solution would be to increase AO dosage, but this, too, is faltered by excessive cost. To remedy this issue, structural modification such that was done by Akpulat et al. (2018) in shortening the commercial 30-mer Eteplirsen into 25-mer AOs could allow cost-efficient administration of higher doses.

One clear advantage of AOs in exon skipping provides the basis for modeling, significantly enhancing its ability to be tailored with, thus improving delivery efficiency and exon skipping efficacy. In this review, 15 out of 23 studies have employed various constructs of different backbones comprising of either phosphorodiamidate morpholino oligomers (PMOs) (Akpulat et al., 2018; Lee et al., 2018), 2′-O-methyl phosphorothioate (2OMePS) AO (Van Putten et al., 2019), 2′-deoxy-2′-fluoro phosphorothioate (2FPS) AO (Jirka et al., 2015), or 2′-O-methoxyethyl oligonucleotide (MOE) (Yang et al., 2013). The results were encouraging, supporting the use of these constructs as possible alternatives. However, it is noteworthy to mention that 2FPS necessitates improvement in the future due to its contradicting results—2FPS had outperformed 2OMePS in the *in vitro* evaluation but was not tolerated in the mdx mouse model (Jirka et al., 2015).

In addition, delivery efficiency was addressed in several studies that introduces the conjugation of these oligomers with peptides, and with the use of delivery agents such as saponins and aminoglycosides (Wang et al., 2018a; Wang et al., 2018b; Wang et al., 2019). While peptide-conjugated PMOs had enabled significant dystrophin overexpression, this approach still requires optimization due to its renal toxicity that prevents escalations in dose-dependent therapies (Roberts et al., 2020). Concordantly, in a study investigating the efficacy of the peptide-conjugated PMO, Pip6a-PMO, death of 5 mdx mice was reported immediately after repeated injections (Blain et al., 2018).

As an amphiphilic naturally occurring compound, saponin has attracted the potential as carriers in drug delivery where studies have reported improved bioavailability (Liao et al., 2021). In exon skipping, the interesting use of saponins as vehicles was first demonstrated and had provided exciting results, digitonin being the most effective. Wang et al. (2018a) reports a 26-fold increase in digitonin-mediated delivery *in vitro* with no obvious cytotoxicity.

### Gene Transfer (AAV and Microdystrophin)

Systemic delivery of functional gene aimed to restore dystrophin expression, most utilizing AAV as a means of mediated delivery, is another interesting chapter broadly studied since it promises high transduction efficiency and stable long-term expression (Kimura et al., 2019; Łoboda and Dulak, 2020). Ideally, recombinant AAVs are best suited for DMD therapy as they exhibit strong tropism toward skeletal muscles (Wasala et al., 2019; Muraine et al., 2020).

Realistically, AAV-mediated gene transfer is impeded by the large size of the dystrophin gene, which is almost three times the capacity of a single AAV virion’s payload (Colella et al., 2018). Realizing such limitations, dual AAVs were adopted along with the notion, from studies in animal models, that functional, though internally deleted dystrophin genes 6–8 kb in size are sufficient for body-wide dystrophin expression (Zhang and Duan, 2012; Duan, 2018). The latter option is seen to be more prominently studied. Specifically, Mendell et al. (2020) demonstrated the intravenous infusion of rAAVrh74. MHCK7. micro-dystrophin in ambulatory boys aged 4 to 7 up to which the results have shown robust transgene expression. In the 3-year follow-up, it was noted that the vector was well tolerated with minimal adverse effects. In addition, Hayashita-Kinoh et al. (2015), in an *in vivo* study of intra-amniotic administration of rAAV-CMV-microdystrophin in canine model, yielded long-term transgene expression and improvements to cardiac function.

In reference to heart failure as the main cause of death in DMD, the potential of this approach in ameliorating heart disease is explored by Bostick et al. (2012). An AAV9-mediated microdystrophin therapy was performed in end-stage models of cardiomyopathy in mdx mice. The study, although did not show significant improvements despite robust expression of micro-dystrophin in treated mice, revealed limitations that served as important findings. In terminally aged mdx mice, the benefit of such an approach is incomparable to what is administered in models of younger age. Indirectly, it answers the question of whether patients in advanced stages of the disease may still benefit from micro-dystrophin therapy, to which the answer is two-pronged: 1) Obviously it would be difficult to induce dystrophin expression in late stages of the disease where fibroadipose tissues would have already overwhelmed cardiomyocytes; however, 2) if the goal is to only alleviate heart disease, such an approach provides promise as a supplement to other interventions. The latter was proposed by Kolwicz et al. (2019) in a novel study employing cardiac troponin T-driven ribonucleotide reductase (RNR) gene transfer. As RNR solely is incapable of inducing statistically significant results, the authors suggested its role as supplemental to microdystrophin treatment.

Besides introducing micro-dystrophin, 2 studies have explored similar AAV-mediated delivery of the beta-1,4-N-acetyl-galactosaminyltransferase 2 (B4GALNT2) gene (formerly known as GALGT2) (Chicoine et al., 2014b; Xu et al., 2019). Xu et al. (2019) reported significantly improved cardiac output following the overexpression of GALGT2; however, Chicoine et al. (2014b) found no significant changes to the DMD phenotypes despite robust expression. It was revealed that there remains a possibility of preexisting antibodies triggered during the study. Pertaining to this obstacle, investigation on whether the removal of AAV binding antibodies would sustain and improve gene expression was necessary. In an attempt by Chicoine et al. (2014a), the removal of suspected AAV binding antibodies had improved gene expression by 4-fold.

### Gene Editing Utilizing CRISPR/Cas9

The application of gene editing, specifically CRISPR/Cas9 system, can accurately correct gene mutations by using either a homology-directed repair (HDR) or nonhomologous end joining (NHEJ) approach (Mollanoori et al., 2021). This approach originates from the bacterial immune system against viruses, in which its discovery was adopted as a tool for gene editing. CRISPR/Cas9 is comprised of a CRISPR-associated endonuclease (Cas) and a guide RNA (gRNA) where both would form a Cas9-gRNA complex known as a ribonucleoprotein directed toward the target DNA. Upon recognition, RNA-guided endonuclease Cas9 produces site-specific double-strand breaks. During the formation of this RNA–DNA heteroduplex, nucleic acid recognition takes place (Palermo et al., 2018). The conformational dynamics within this heteroduplex is influenced by the bi-lobed architecture of the Cas9 protein that consists of recognition lobes (REC1-3) that mediate binding and a nuclease lobe with domains RuvC and HNH responsible in controlling cleavage activity (Nishimasu et al., 2014; Jiang et al., 2015; Sternberg et al., 2015).

With the capacity to permanently edit specific genes, numerous studies utilizing CRISPR/Cas9 to correct DMD mutation have been performed in recent years. Gene editing with regards to DMD is at large focused at generating an in-frame mutation similar to that of BMD (Chemello et al., 2020). Lattanzi et al. (2017) demonstrated restoration of wild-type dystrophin following the removal of a duplicated exon 2 by a single administration. Remarkably, no off-target effects were detected, although it was only expressed in 5% of total cell population. While delivery efficiency was not addressed, it remains one of the many challenges of the CRISPR/Cas9 approach (Uddin et al., 2020). To drive better delivery, AAV is considered as a vehicle such that is performed by Koo et al. (2018) taking into advantage the tropism exhibited by AAV9. However, relying solely on AAV delivery poses more challenges such as preexisting immunity and possibilities of off-target effects from sustained expression (Lee et al., 2017). In spite of that, AAV vectors are still considered the key delivery vehicle due to their high efficiency (Xu et al., 2019).

As proposed by Lee et al. (2017), gold-conjugated delivery in lieu of viral delivery vehicles prompted HDR repair restoring wild-type dystrophin; however, they are still observing its cytotoxicity. Significant elimination of potential toxicity of mediated delivery was seen in the use of extracellular nanovesicles where little to no off-target cleavage was observed while inducing permanent exon 45 deletion (Gee et al., 2020). The approach is nontoxic, although it was noted that the expression of its single guided RNA was driven by HIV-1 Tat, posing the risk in the event of nonspecific incorporation. It was also reasoned that the limited capacity of AAV may hinder the delivery of the various components of CRISPR/Cas9, but ultimately proven that payload limitation could be resolved using significantly smaller protein such as SaCas9 (Duchêne et al., 2018).

One of the main concerns on gene editing via CRISPR/Cas9 lies in its off-target effects with frequencies of 50% or more (Zhang et al., 2015; Uddin et al., 2020). To remedy this, modifications were suggested such as desensitization of the REC3 as witnessed in the variants of Cas9 (Valkuskas et al., 2018). As an extension to the conformational dynamics mentioned earlier, it was revealed that REC participates in subsequent conformational activation of the highly flexible HNH domain (Sternberg et al., 2015). To prevent the possibilities of off-target genome editing, alterations to REC3 prevent downstream conformation in the HNH domain when bound to off-targets (Chen et al., 2017; Han et al., 2020). This was also demonstrated in a study by Koo et al. (2018) utilizing highly specific CjCas9.

### From Drug Prescription to Gene-Based Therapies

Conventionally, the gold standard of DMD drug therapy is the administration of glucocorticoid steroids, either prednisone or deflazacort, that are routinely prescribed to prevent inflammation-associated tissue damage and, to an extent, effectively delay disease progression (Kharraz et al., 2014; Zhang and Kong, 2021). Glucocorticoids, as demonstrated by Morrison-Nozik et al. (2015), were able to enhance muscle strength and provide ergogenic effects. Furthermore, it was established that the downstream cascade after glucocorticoid receptor-mediated activation of Kruppel-like factor 15 (KLF15) mitigates dystrophic severity of the disease (Morrison-Nozik et al., 2015; Ahlskog et al., 2019). Although the findings illuminate the relationship between glucocorticoids and KLF15, and possibly the potential to improve DMD pathology in the event of overexpression, it was also noted that the downstream targets of this mechanism are insufficient (Chen et al., 2000; Morrison-Nozik et al., 2015).

Prolonged glucocorticoid treatment induces adverse effects including delayed puberty, adrenal insufficiency, cataracts, osteoporosis, and obesity (Zhang and Kong, 2021). To date, the most widely used regimen for DMD are corticosteroids routinely prescribed along with proper patient management in delaying the disease progression (Colella et al., 2018). While groundbreaking research and clinical trials in recent years have made interesting discoveries, it is still evident that there remains no cure for DMD yet. Now, with new platforms that provide intense research in gene-based strategies, the capacity of such approach in curing DMD demonstrates great potential. In this systematic review, we aimed to provide an overview of the most recent strategies in research, and highlighting the success, potential, and obstacles in each approach.

From our initial search until the inclusion of eligible articles, it is noticed that exon skipping remains fairly popular in research. This is evidenced by the indefinite avenues for engineering PMO constructs by attaching different backbones and conjugating the oligomers with various molecules that could enhance delivery and exon skipping efficiency. As discussed earlier pertaining to overcoming obstacles in AAV-mediated interventions, the incorporation of bioactive molecules in PMOs as carriers may significantly enhance exon skipping. In addition, antisense RNA sequences such that of U7snRNA were found to be exceptionally feasible considering their minimal efficacious dose establishing near full-length dystrophin restoration in both skeletal and cardiac muscles (Simmons et al., 2021).

Despite that, persisting challenges in exon skipping include chemical-dependent toxicity in the case of conjugated PMOs and short-lived effect *in vivo*. Several studies have already addressed the former obstacle by minimizing toxicity, while the latter, though interestingly preserved when delivered with a viral vector, posed another challenge with regard to AAV-mediated efficiency. As thoroughly discussed in the results, there are possibilities that viral vector-mediated approaches could be rendered ineffective due to preexisting humoral immunity (Giles et al., 2020). It is complicated since the full extent of such an immune response may not be fully reflected in *in vivo* studies employing DMD models as noted by Chicoine et al. (2014b). Surprisingly, regardless of such risk, there are still ongoing studies because AAV-mediated exon skipping provides the highest efficacy in dystrophin recovery. In this critical evaluation of gene-based therapies, it is eminent that with every proposed solution comes two obstacles. It is also necessary to underline that the objective of most therapies is to effectively dial down the severity of DMD to that of BMD’s, and this too is faced with challenges. In our view, there are still avenues to which future research could expand on in CRISPR/Cas9. Several risks and challenges were already addressed with proposed modifications as previously mentioned. Owing to its relatively cheap cost and high specificity, it is opined that CRISPR/Cas9, though relatively new, may provide better opportunities in DMD treatment.

**FIGURE 2:**
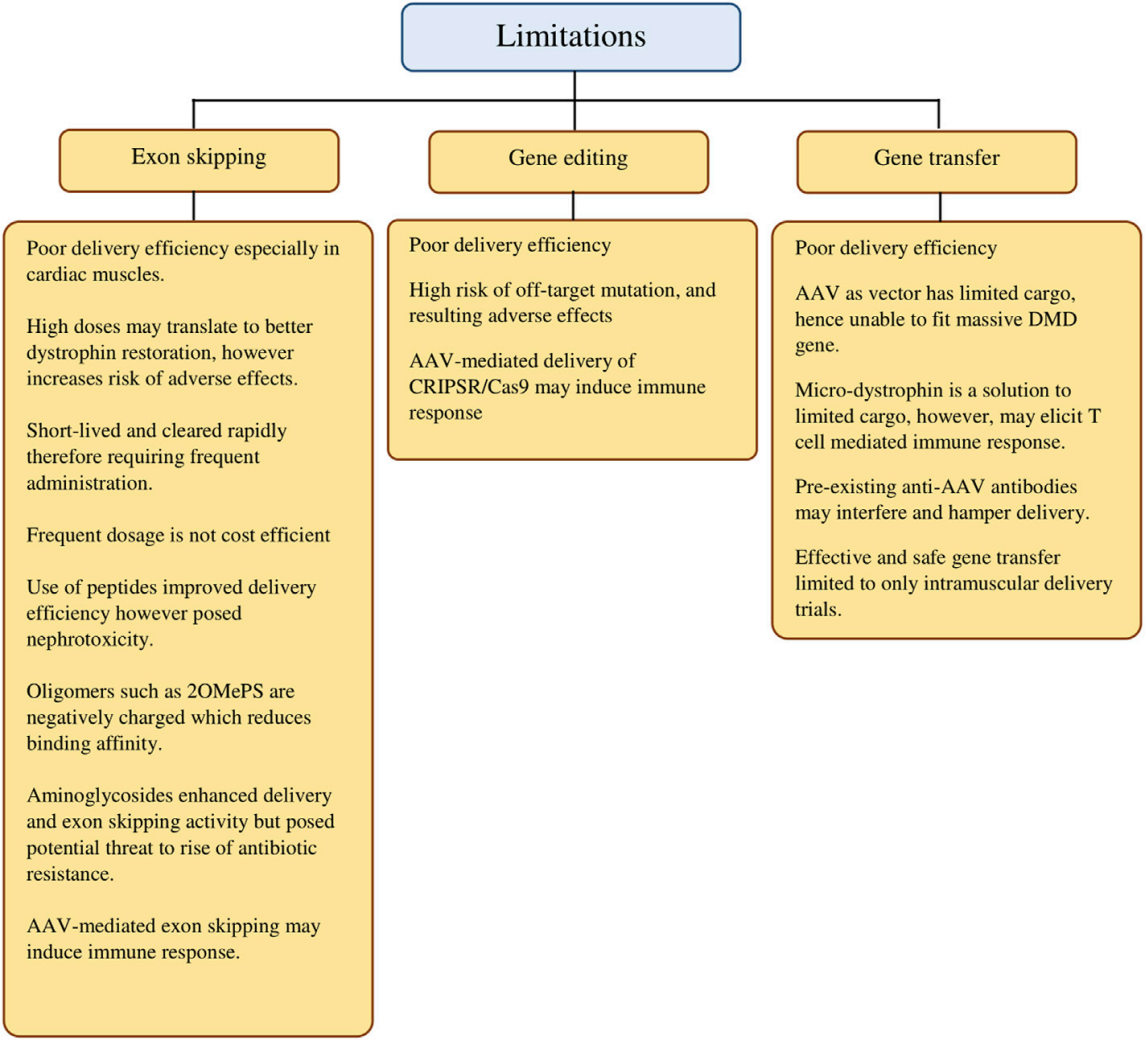
Current limitations of gene-targeted therapeutic strategies in DMD treatment.

In summary, we provide a panoramic overview of the gene-based approaches in DMD treatment in the last decade to highlight individual progress and notable results while remaining critical in evaluating each approach. More importantly, we have highlighted the current limitations (Figure 2) and noted on alternatives evidenced by findings from the selected studies that were elaborated earlier. In addition, we intend to provide a concise point of reference that studies in the coming years may revisit the current shortcomings with more effective solutions.

### Limitations of the Study

In this review, we recognize our limitations. We acknowledge the high number of articles that are deemed relevant. This is also due to our relatively large focus—gene-based interventions and therapies. That said, we have attempted to minimize the said limitation by having three authors to independently screen, select, and extract data, and subsequently also performing a risk-bias assessment on the selected articles. Having to include only original research articles of such focus, we acknowledge that at the time of writing, more recent studies are performed or are in the process of publication, which meant that key findings might have been missed out.

## Future Research Directions and Perspectives

Irrespective of the approach, the continuous advancement of technology and better grasp of novel techniques may provide answers to questions today with regard to improving overall efficacy and efficiency while not discounting the need to prioritize safety by scrutinizing possible adverse effects in long-term exposure of any of these gene-based approaches. With respect to CRISPR/Cas9, there have been already promising results in strengthening further research efforts in this direction. In line with that, we hope that the controversies surrounding the use of CRISPR/Cas9, especially the public’s view, will be cleared off by outcomes of future clinical trials. Nevertheless, further work on improving delivery efficiency, feasibility, and safety of other approaches may also bring promise to other gene-based approaches. To this, we are looking forward to efforts in elucidating immunological responses toward various vector or vehicle-mediated delivery in microdystrophin gene delivery and AOs, performed both *in silico* and *in vivo* in larger samples of mice and nonhuman primates.

In addressing DMD as a whole, it is of paramount importance to provide better diagnosis of DMD and the need of discovering specific markers for accurate assessment. Proper assessment, in this sense, also meant the inclusion of data in real time, however requiring updated and routine data collection from various healthcare sources globally to provide better understanding in patient management and clinical progression of the disease. Stressing on the mutational spectrum of DMD, we hope that rigorous in-depth analysis can account for a personalized medicine approach for inter-individual variability through means of identifying single nucleotide polymorphisms (SNPs). At the same time, we look forward to interdisciplinary research focused on the transplantation of patient-derived stem cells corrected either by exon skipping or the CRISPR/Cas9 system.

In conclusion, we hope that future research and clinical trials may provide a definite cure, if not better approaches, that will ultimately improve the quality of life for DMD patients.

## Data Availability

The original contributions presented in the study are included in the article/Supplementary Material, further inquiries can be directed to the corresponding author.
