# Supplementary material for: A Decade of Progress in Gene Targeted Therapeutic Strategies in Duchenne Muscular Dystrophy: A Systematic Review
Source: Front Bioeng Biotechnol. 2022 Mar 23;10:833833. doi: 10.3389/fbioe.2022.833833 (PMC8984139; doi:10.3389/fbioe.2022.833833)
Supplement: Supplementary file 2 [file Table2.DOCX]

**S2 Appendix: Search strategy**

| Gene targeted therapeutic strategies for Duchenne Muscular Dystrophy | |
| --- | --- |
| Keywords | (DMD) AND ((Gene) OR (Genetic)) AND ((Therapy) OR (Treatment)) |
| Inclusion criteria | Articles within 10 years, ONLY English research articles relevant to the topic of interest; in-vivo, in-vitro, and RCTs |
| Exclusion criteria | Reviews, proceedings, commentaries, reports, guidelines  (DMD) AND ((Gene) OR (Genetic)) AND ((Therapy) OR (Treatment)) NOT (review) |

**Database: ScienceDirect**

| Keyword | | | Inclusion | | Exclusion criteria considered | Relevant articles |
| --- | --- | --- | --- | --- | --- | --- |
| (DMD) | (DMD) AND ((Gene) OR (Genetic)) | (DMD) AND ((Gene) OR (Genetic)) AND ((Therapy) OR (Treatment)) | Article within 10 years (2012-2021) | Research articles only |  |  |
| 34,730 | 12415 | 9148 | 4510 | 1620 | 548 | 40 |

**Database: PubMed**

| Keyword | | | Inclusion | | Exclusion criteria considered | Relevant articles |
| --- | --- | --- | --- | --- | --- | --- |
| (DMD) | (DMD) AND ((Gene) OR (Genetic)) | (DMD) AND ((Gene) OR (Genetic)) AND ((Therapy) OR (Treatment)) | Article within 10 years (2012-2021) | Research articles only |  |  |
| 9,175 | 4914 | 2302 | 1566 | 45 | 17 | 2 |

**Database: ProQuest**

| Keyword | | | Inclusion | | Exclusion criteria considered | Relevant articles |
| --- | --- | --- | --- | --- | --- | --- |
| (DMD) | (DMD) AND ((Gene) OR (Genetic)) | (DMD) AND ((Gene) OR (Genetic)) AND ((Therapy) OR (Treatment)) | Article within 10 years (2012-2021) | Research articles only |  |  |
| 97,151 | 30470 | 25140 | 21426 | 4878 | 395 | 37 |
