# Supplementary material for: A Decade of Progress in Gene Targeted Therapeutic Strategies in Duchenne Muscular Dystrophy: A Systematic Review
Source: Front Bioeng Biotechnol. 2022 Mar 23;10:833833. doi: 10.3389/fbioe.2022.833833 (PMC8984139; doi:10.3389/fbioe.2022.833833)
Supplement: Supplementary file 3 [file Table3.DOCX]

| **Exon skipping** | | | | | | |
| --- | --- | --- | --- | --- | --- | --- |
| References | Aim | Therapeutic product | Type of gene therapy | Additional information | Findings | Conclusion and correlation to DMD therapy |
| Akpulat, *et al*., 2018 [47] | To examine the exon-skipping efficacy of shorter 25-mer PMOs | Three 25-mer PMOs of Eteplirsen;  Etep-Upstream, Etep-Middle, and Etep-Downstream | Exon-skipping | An experimental quantitative exon-skipping assay on exon-52-deleted H2K-*mdx*52 mouse myoblast cell line and *mdx*52 dystrophin-null mouse model | 1. Efficacy of Etep-Upstream comparable to Eteplirsen 2. Shorter 25-mer PMOs is able to establish dystrophin-expressing myofibres. 3. Target sequence and PMO interaction is dose-dependent; directly proportional, however, non-linear | 25-mer Etep-Upstream can induce similar dystrophin levels as 30-mer-PMO Etep. |
| Blain, *et al*., 2018 [48] | To investigate the efficacy of the peptide-conjugated PMO, Pip6a-PMO for restoration of cardiac dystrophin and functional rescue in DMD mice | Pip6a-PMO | Exon-skipping | An *in vivo* study employing a peptide-conjugated PMO on male *mdx,* *C57BL10* and *Cmah-/-mdx* mouse | 1. Significant dystrophin restoration in all cardiomyocytes  2. Improved cardiac function.  3. However, death is reported possibly due to cumulative toxic effects at higher treatment dose | Positive effect of P-PMO treatment, however, future optimization on structure and clinical improvement on toxicity is required. |
| Domenger, *et al*., 2018 [49] | To perform clinical trial using rAAV-mediated uridine-rich small RNA (UsnRNA) in DMD treatment | rAAV-U7snRNA-E53 | Exon skipping | Study was performed on human primary myoblasts and human primary hepatocytes on exon skipping efficacy and to study occurrence of off-target effects. | 1. Therapeutic product expressed in both tested human cells.  2. U7-E53 RNA triggered specific exon skipping on DMD target. | Effective exon skipping without inducing notable deregulation of transcripts in human cells. Off-target impacts are negligible. |
| Echigoya, *et al*., 2017 [50] | To evaluate the effectiveness of morpholino-based antisense oligonucleotides (AO) for exon 51 using systematic screening method involving in silico, in vitro, and in vivo tests. | Morpholino antisense oligonucleo-tides (AO) | Exon skipping | *In silico* analysis was performed to predict exon skipping efficiency of the designed AO sequences, in which the selected AOs will be tested in human derived skeletal muscle cells (in vitro) and in *Dmd* exon 52-deficient *mdx52*, and wild-type C57BL/6J mice (in vivo). | 1. Designed AOs exhibit better efficiency, 12-fold and 7-fold increase, in exon 51 skipping compared to eteplirsen sequence.  2. Initial 5’ site of exon 51 is important for inducing skipping. | AOs designed for exon 51 skipping was found to be potentially more effective than the 30-mer eterlipsen, with consistent expression of rescued dystrophin. |
| Goemans, *et al*., 2016 [51] | To study the long-term efficacy, safety, and pharmacokinetics of Drisapersen. | Drisapersen | Exon skipping | A Phase 1/2a open-label dose-escalation study in 12 male DMD subjects for 188-weeks. | 1. 6-minute walking distance (6MWD) test showed improvement.  2. Muscle strength decreased over 177 weeks. However, inter-individual variability is high, hence, clear overall trend is difficult to be determined.  3. Mild adverse effects over 177 weeks. | Drisapersen is well tolerated, however, presents commonly prominent injection-site reactions. Results are promising, but drisapersen has not been approved by the FDA in DMD treatment. |
| Jirka, *et al*., 2018 [52] | To investigate the efficiency of cyclic peptides in delivery of antisense oligonucleotides, and exon skipping *in vitro* and *in vivo*. | Cyclic-peptide conjugated 2’-O-methyl phosphorothi-onate antisense oligonucleoti-des (CyPep-2OMePS AON) | Exon skipping | A study on the use of muscle-homing 7-mer cyclic peptide conjugated with 2OMePs AON in systemic delivery and exon 23 skipping activity *in vitro* (human control myotubes) and *in vivo* in *mdx* mouse | 1. Out of 12 candidate cyclic peptides screened, 2 peptides, CyPep6 and CyPep10 were selected for further studies.  2. CyPep6- and CyPep10-conjugated 23AON reported 2-fold increase of AON in skeletal muscles, and 3-fold increase in diaphragm muscle and heart.  3. Increased exon skipping in CyPep10-23AON.  4. Dystrophin expression was, however, very low. | CyPep10 is deemed a potential candidate for AON delivery, however, extended studies on long-term treatment effects especially on restoration of dystrophin expression is required in the future. |
| Lee, *et al*., 2018 [53] | To demonstrate efficacy of *in vitro* exons 45-55 skipping in myotubes converted from *DMD* patient fibroblast using antisense PMOs | 30-mer antisense oligomer | Exon skipping | A novel study on the use of cocktail of PMOs to induce exons 45-55 skipping in myotubes transdifferentiated from patient fibroblast cells | 1. Exons 45-55 skipping was demonstrated with in-frame link between exons 44 to 56.  2. Dystrophin rescue expressed in cells. | Exons 45-55 skipping was demonstrated whilst validating the use of transdifferentia-ted fibroblast cells. |
| Lu-Nguyen, *et al*., 2017 [54] | To study the efficacy of PMO conjugated with B peptide (BPMO) in inducing exon skipping of myostatin and dystrophin restoration. | BPMO-M23D and BPMO-MSTN | Exon skipping | A study employing intravenous systemic delivery of BPMO-M23D and BPMO-MSTN targeting exon 23 of dystrophin gene and exon 2 of myostatin gene in *mdx* mice | 1. Efficient skipping of exon 23 and 2.  2. BPMO-M23D stimulates body-wide dystrophin rescue.  3. Dystrophic features in mdx mice were ameliorated. | Systemic delivery of antisense BPMOs had demonstrated body-wide dystrophin expression and rescue, and myostatin inhibition with pathological features ameliorated. |
| (Pires, *et al*., 2016 [55] | To evaluate the ability of locked nucleic acid (LNA)-modified antisense oligonucleotides to induce exon 51 skipping. | LNA-modified splice switching antisense oligonucleotides (LNA-modified SSO) | Exon skipping | A study on the use of LNA-modified antisense oligonucleotides to induce exon 51 skipping and dystrophin restoration in DMD patient-derived myoblast cells. Approach was compared with AO51 2’-O-methyl-phosphorothioate SSO. | 1. 16-mer LNA-modified SSO efficiently induced exon skipping and wild-type dystrophin restoration, demonstrated to be more effective than AO51.  2. Expression and localization of dystrophin in the sarcolemma was observed. | Effective exon 51 skipping demonstrated by 16-mer LNA-modified SSO with restoration of truncated dystrophin protein in patient-derived myoblast cells. |
| Van Putten, *et al*., 2019 [56] | To study exon various aspects influencing exon skipping efficiency of 2OMePS AONs in nonclinical animal studies. | 2OMePS AONs | Exon skipping | 2OMePS AONs were administered to study: impact of age and treadmill running in *mdx/BL10* mice; exon skipping efficiency in *D2-mdx* mouse; and survival of *mdx-utrn-/-* mice. | 1. No significant difference in exon skipping efficiency and dystrophin expression between old and younger mice.  2 Minimal increase, however, non-significant difference in exon skipping levels in treadmill-exercised mice.  3. Survival of *mdx-utrn-/-* mice were slightly increased. | Nonclinical experiments were performed, which their intentions were to investigate questions raised with regards to clinical trials employing AONS. |
| Vila, *et al.* 2019 [57] | To study the effects of chronic PMO treatment on skeletal muscle pathology. | PMO | Exon skipping | An *in vivo* study on the effects of chronic PMO treatment on skeletal muscle pathology, and the possibilities of eliciting dystrophin-specific autoimmune response in male *mdx* mouse after *de novo* dystrophin expression | 1. Significant inflammation and macrophage infiltration after exon skipping.  2. PMO treatment, even in low doses, triggered generation of dystrophin-reactive antibodies.  3. Elevated T-cell response. | De novo dystrophin expression can trigger cell-mediated and humoral immune responses, which reflects on the variable success of exon-skipping therapies. |
| Wang, *et al*., 2018a [58] | To investigate in vitro and in vivo delivery efficiency of PMOs with saponins. | Saponins: Digitonin, Tomatine, and Glycyrrhizin. | Exon skipping | A novel study on the efficiency of three commercially available saponins as vehicles to deliver antisense PMOs *in vitro* in C2C12E50 and C2C12E23 myoblasts, and *in vivo* in *mdx* mice. | 1. Digitonin and Tomatine improved in vitro exon skipping in both myoblast cell types.  2. Digitonin-mediated delivery of PMO for exon 23 skipping had 7-fold enhancement.  3. Local and systemic delivery of PMO were increased with integration of saponins. | Saponins, especially digitonin had improved delivery of PMO up to 7-folds with no obvious cytotoxicity observed. |
| Wang, *et al*., 2018b [59] | To investigate in vitro and in vivo delivery of 2’-OMePS with saponins as the delivery vehicle. | Saponins: Digitonin, Tomatine, Lanoxin , and Glycyrrhizin. | Exon skipping | A novel study on the efficiency of in vitro and in vivo delivery of 2’-OMePS in C2C12E50 myoblasts and in dystrophic *mdx* mice with the use of saponins. Efficiency of delivery was compared with Lipefectamine 2k (LF-2K) as control vehicle. | 1. Digitonin- and Tomatine-mediated delivery was 26- and 13- fold higher.  2. Saponins used had lower toxicity than LF-2k even at higher doses.  3. *In vivo* delivery showed an almost 10-fold increase in efficiency. | Among the tested saponins, digitonin as the vehicle demonstrated significant increase in 2’-OMePS delivery efficiency that enabled enhanced exon skipping activity. |
| Wang, *et al*., 2019 [60] | To investigate the efficiency of aminoglycosides as PMO delivery-enhancing agents. | Aminoglycosid-es (AG): Amikacin, Apramycin sulfate, Kanamycin A sulfate, Neomycin sulfate, Paromomycin sulfate, Streptomycin sulfate, Sisomycin sulfate, and Gentamycin sulfate. | Exon skipping | A study on employing AGs for *in vitro* and *in vivo* delivery of PMOs in C2C12E50 and C2C12E23, and in *mdx* mice. Cytotoxicity of the AGs were also evaluated. | 1. AGs exhibited lower cytotoxicity.  2. Efficient *in vitro* AG-mediated delivery in both myoblasts and myotubes.  3. Up to 6-fold increase in dystrophin expression with *in vivo* Kanamycin A sulfate- and Sisomycin sulfate- mediated delivery.  4. Increased dystrophin expression following systemic delivery.  5. No obvious toxicity and pathological changes in organs of treated mice. | Antisense PMO-mediated exon skipping activity was improved with the use of AGs as its vector. |
| Watanabe, *et al*., 2018 [61] | To perform pharmacological study on PMO, NS-065/NCNP-01 and its exon skipping efficiency. | NS-065/NCNP-01 | Exon skipping | A study to assess sequence-dependent effects of AOs, and non-clinical pharmacology study of PMO, NS-065/NCNP-01 and *in vitro* exon 53 skipping in DMD patient derived cells | 1. NS-06/NCNP-01 exhibited highest level of exon 53 skipping among 25 tested PMOs.  2. Exon skipping activity and dystrophin expression sustained for two weeks after single transfection. | Administration of NS-065/NCNP-01, according to results of the study suggests potential therapeutic effect. |
| Yang, *et al*., 2013 [62] | To study the effect of 2’-O-methoxyethyl oligonucleotide (MOE) in exon skipping and dystrophin restoration. | MOE | Exon skipping | A study employing three MOE designs with different backbones and lengths, and their effects in exon 23 skipping *in vitro* and *in vivo*. | 1. 25-mer MOE with a phosphorothioate (MOE25(PS), exhibited highest exon skipping efficacy with dystrophin fibers expressed in tibialis anterior muscle of *mdx* mice.  2. Phosphorotioate backbone showed superior effectivity in inducing exon skipping. | The potential of MOE PS in effectively inducing exon skipping and dystrophin expression was demonstrated, thus suggesting MOE PS as an alternative treatment option. |
| Betts, *et al*., 2012 [63] | To investigate the exon skipping efficacy of derivatives of Pip5e-PMO, and how structural changes affect its activity. | Pip6-PMO | Exon skipping | A study on the development of potential derivatives of Pip5e-PMO in exon skipping and dystrophin restoration. Derivatives were demonstrated *in vitro* in mdx mouse myotubes and *in vivo* in mdx mice. | 1. Pip6-PMOs, a derivative, demonstrated improved exon skipping and dystrophin production in various muscles.  2. Structure of hydrophobic core of the derived PMO was found significant.  3. Alterations to arginine reduced dystrophin expression. | Improved exon skipping activity was demonstrated while the PMO’s hydrophobic core suggests its significance in heart dystrophin production. |
| Bish, *et al*., 2012 [64] | To demonstrate the efficacy of rAAV6-mediated exon skipping in golden retriever muscle dystrophin (GRMD) model | rAAV6-mediated delivery of snRNA | Exon skipping | A novel *in vivo* study on percutaneous transendocardial delivery of rAAV6 as vector to transport a modified U7 small nuclear RNA (snRNA) to induce exon skipping and dystrophin restoration in GRMD canines | 1. High-dose rAAV6 treatment showed high exon skipping efficiency, with dystrophin protein detected in regions of the heart.  2. Improved cardiac function and reduced fibrosis in treated canines. | Method of delivery and vector was proven to be safe and effective in dystrophin restoration. |
| Cazzella, *et al*., 2012 [65] | To investigate dystrophin rescue ability of U1 snRNA-antisense constructs and its potential to recover dystrophin-nNos (Dys-nNos) pathway. | U1 snRNA-antisense constructs, #1 to #8 | Exon skipping | A study assessing the effectivity of several U1 snRNA-antisense constructs for *in vitro* dystrophin rescue and its impact on myogenic differentiation in human DMD myoblasts | 1. Among tested constructs, #3 and #4 exhibited highest dystrophin rescue.  2. Dystrophin rescue results in reactivation of Dys-nNOS pathway | U1 snRNA backbone as constructs for antisense molecules considerably restored dystrophin via Dys-nNos pathway reactivation. |
| Jirka, *et al*., 2015 [66] | To study the exon skipping efficacy of 2’-deoxy-2’-fluoro (2F) AO | 2’-F phosphorothioate (2FPS) | Exon skipping | The study comparatively assesses efficacy of 2FPS and 2’-O-Me phosphorothioate (2OMePS) to study the ability of 2FPS in inducing *in vitro* and *in vivo* exon skipping in human and mouse myoblast, and in *mdx* mouse. | 1. *In vitro* evaluation showed that 2FPS performed better than 2OMePS in exon 23, 45, and 53 skipping activity, however, exon skipping activity of 2FPS *in vivo* was not detected.  2. 2FPS failed to induce exon skipping in vivo, and dystrophin restoration.  3. Both 2FPS and 2OMePS did not induce toxic effects. | The contradicting efficiency of 2FPS in mdx mouse model when reflected with its activity in cell cultures suggesting that 2FPS treatment was not tolerated, which outcome of study does not support clinical application of 2FPS antisense oligonucleotide. |
| Malerba, *et al*., 2012 [67] | To study the efficacy of an alternative PMO chemistry in myostatin knockdown via *dmd* exon skipping. | Octaguanidine-conjugated PMO, Vivo-PMO | Exon skipping | The study employs the use of Vivo-PMO to induce *in vivo* exon skipping in C57BL10 mice via intramuscular administration, and comparatively analyzes its efficacy with B-PMO singly. Efficacy of Vivo-PMO and B-PMO co-injection was also studied. | 1. Administration of single dose induced significant increase in muscle mass.  2. Single intramuscular administration of B-PMO also induced increase in muscle mass.  3. Co-injection demonstrated improved exon-skipping and dystrophin expression | The approach of a dual antisense combination therapy in inducing myostatin exon skipping shows significant potential. |
| Simmons, *et al*., 2021 [68] | To investigate the efficacy of an AAV-based U7snRNA exon-skipping approach, and establish minimal efficacious dose for human clinical trial. | scAAV9.U7.ACCA | Exon skipping | The study assesses the efficacy of in vivo administration of U7snRNA, with a dose-escalation study in both intramuscular and systemic administration to establish its therapeutic range in Dup2 and C57BL/6 (Bl6) mice. | 1. In intramuscular administration, dystrophin expression at the sarcolemma is found to be directly proportional to dosage.  2. Functional rescue in Dup2 was greater than in Bl6, however remains more susceptible to injury that the latter.  3. In systemic administration, increased exon 2 skipping in higher doses.  4. Expression of dystrophin positive fibers and signaling intensity is directly proportional to dosage, however, it signal intensity was less intense at the sarcolemma. | Feasibility of the approach is demonstrated, with a minimal efficacious dose of 3 x 10^13^ vg/kg establishing expression of near full-length dystrophin in skeletal and cardiac muscles. |
| Wang, *et al*., 2013 [69] | To study the potential of polyethylenimine-modified pluronic (PCM) copolymers in PMO delivery. | PCM-mediated PMO | Exon skipping | A series of PCM polymers, PCM-01 to PCM-14 were employed for the delivery of PMO *in vitro* and *in vivo* in C2C12E50 cells and *mdx* mice respectively. | 1. Toxicity of PCM was found to be dose dependent, and is directly proportional to degree of its hydrophobicity.  2. *In vitro* exon skipping was enhanced, maximum expression of GFP reporter was achieved at 5 μg and 10 μg.  3. Drastic increase in dystrophin positive fibers in treated muscles via intramuscular administration.  4. Three polymers inducing highest dystrophin expression were selected for in vivo systemic administration, expressing low toxicity and significant exon skipping in cardiac muscles. | The study provides evidence suggesting high potential of integrating bioactive molecules that enhances the pharmacological application of PMO. |
| **Gene editing** | | | | | | |
| Chen, *et al*., 2021 [70] | To evaluate potential of novel CRIPSR-mediated large-scale excision in reframing DMD mutation | CRISPR/Cas9 & CRISPR-Cas12a system | Gene editing | A study employing CRISPR-Cas9 and CRISPR-Cas12a for in vivo genome editing in patient-derived xenograft DMD mouse model. | 1. High efficiency in restoring dystrophin protein expression.  2. CRISPR-Cas12a shows same efficiency as CRISPR-Cas9.  3. Restored dystrophin was functional. | CRISPR-Cas9 system demonstrated in vivo genome editing restored dystrophin in human muscle cells. |
| Duchenne, *et al.*, 2018 [71] | To examine the therapeutic potential of CRISPR/SaCas9 for DMD patients with different mutations in the DMD gene. | CRISPR-SaCas9 system | Gene editing | Study was performed on *hDMD/mdx* mouse with SaCas9 delivered with a pair of sgRNAs in AAV9 vectors. | 1. Combinations of two pairs of sgRNAs with the highest precision resulted in formation of hybrid exons 47-58.  2. Low frequency of off-target mutations, however extensive characterization was opined.  3. In vivo restoration of DMD reading frame and dystrophin expression in mouse model heart. | CRISPR/SaCas9 displayed potential in restoration of DMD reading frame and dystrophin expression. |
| Gee, *et al*., 2020 [72] | To examine the efficiency of extracellular nanovesicles for in vivo delivery of CRISPR-SpCas9 RNP and sgRNA. | NanoMEDIC, an extracellular vesicle | Gene editing | A study on the use of NanoMEDIC as an extracellular vesicle in delivery in HEK293T cells and DMD patient iPSC, in vitro, and C57BL/6J mice, in vivo. | 1. NanoMEDIC capable of inducing exon 45 deletion, and dystrophin expression in iPSCs of DMD patient.  2. Off-target cleavage was nearly eliminated.  3. Two sgRNAs were independently delivered, demonstrating multiplexed genome editing. | NanoMEDIC was found capable of inducing permanent exon skipping in a luciferase reporter mouse and in *mdx* mice. |
| Koo, *et al*., 2018 [73] | To investigate the potential of AAV mediated deliver of CjCas9 in correcting disrupted *Dmd* reading frame and dystrophin restoration. | CjCas9, derived from *Campylobact-er jejuni* | Gene editing | A study on the use of CjCas9 and its sgRNA, packaged into AAV serotype 9 vector, to induce *Dmd* frameshift correction via non-homologues end joining in Dmd knockout and C57/BL6J mice | 1. CjCas9 is highly specific.  2. AAV2/9-CjCas9 delivery induced dystrophin expression in TA muscles and myofibers at the sarcolemma.  3. Treated muscles expressing dystrophin had increase in specific maximal force. | Highly specific and efficient CjCas9 mediated correction of the DMD frame shows great treatment potential |
| Lattanzi, *et al*., 2017 [74] | To demonstrate dystrophin restoration by correcting exon 2 duplication using a single CRISPR/Cas9 system. | CRISPR/Cas9 system | Gene editing | A study on the use of a single gRNA guided CRISPR/Cas9 system in restoring exon 2 duplication in immortalized *DMD* myoblasts to restore synthesis of wild-type dystrophin. | 1. Intense dystrophin expression, however, only occurring in 5% of total cell population.  2. High on-target activity, with no off-target effect detected. | Duplicated exon 2 was removed with just one gRNA, demonstrating editing efficiency of this novel approach. |
| Lee, *et al*., 2017 [75] | To study the efficacy of in vivo gold nanoparticles conjugated delivery of Cas9 ribonucleoprotein and donor DNA. | CRISPR-Gold vehicle | Gene editing | A study on the use of CRISPR-Gold vehicle for in vivo delivery, employing cationic properties of poly(N-(N-(2-aminoethyl)-2-aminoethyl) aspartamide) (PAsp(DET) in correcting gene mutation via homology-directed repair. | 1. CRISPR-Gold is dependent on caveolae/raft-dependent endocytosis, suggesting significance of PAsp(DET).  2. CRISPR-Gold able to target CXCR4 in wide range of cells.  3. Point mutation in *mdx* mouse was corrected, with dystrophin expressed.  4. Mutated dystrophin gene in *mdx* mice was corrected to wild-type sequence.  5. Minimal off-target DNA damage. | CRISPR-Gold was demonstrated to be able to deliver Cas9 protein gRNA, and donor DNA, in vitro and in vivo, with the ability to edit genes via HDR and potential to restore wild-type sequences in mutated genes. |
| Lopez, *et al*., 2020 [76] | To demonstrate homology directed repair (HDR) – mediated gene editing with CRISPR/Cas9 and TALEN in GRMD myoblasts. | CRISPR/Cas9 system and TALEN | Gene editing | A novel study employing paired CRISPR/Cas9-TALEN to perform HDR-mediated gene editing *in vitro* and *in vivo* of GRMD myoblasts and GRMD dogs. | 1. Successful in vitro gene correction in DNA and RNA, however, no significant changes in dystrophin expression.  2. Positive protein expression from *in vivo* administration of sgRNA A- and sgRNA B-guided CRISPR/Cas9.  3. Significant improvements on pathological features of DMD were not seen. | The approach showed lack of HDR repair at protein level which prompted necessary modifications on methods in future studies. |
| Iyombe-Engembe, *et al*., 2016 [77] | To examine the potential of CRISPR-induced deletion (CinDel) in correcting the DMD gene. | CRISPR/Cas9 system | Gene editing | CinDel employs the use of two gRNAs to target and induce deletion following precise DSB, thus restoring the DMD reading frame. This study is demonstrated *in vitro* in 293T cells and *in vivo* in hDMD/*mdx* mice. | 1. *In vitro* demonstration of CinDel technique removed a portion of the DMD gene.  2. gRNA pair, gRNA2-50/2-54 induced large exon deletion *in vivo*, thus restoring reading frame in the DMD gene, resulting in expression of myotube and truncated dystrophin. | Direct restoration of the DMD was demonstrated, however, further experimentation required for fine tuning. |
| Gene transfer | | | | | | |
| Bowles, *et al*., 2012 [78] | To perform clinical trial using chimeric AAV capsid variant derived from rational design strategy | AAV2.5 | Gene transfer | A first clinical trial; randomized double-blind placebo-controlled phase I clinical study with 15-year follow-up on 6 patients | 1. No cellular immune response towards capsid.  2. Low minidystrophin transgene expression. | Vector is safe, well tolerated with no vector-related adverse effects.  Opens possibility to AAV vector customization to best suit clinical objective. |
| Kolwicz, *et al*., 2019 [79] | To evaluate whether enzyme ribonucleotide reductase (RNR) overexpression can ameliorate cardiac dysfunction. | rAAV6-cTnT455-RNR | Gene transfer | This novel study employs AAV6-mediated cardiac troponin T-driven RNR (rAAV6-cTnT455-RNR) to induce RNR overexpression *in vivo* in *mdx* mice. Efficacy of the approach is compared with AAV6-mediated CK8-driven microdystrophin (rAAV6-CK8-μDys), and rAAV6-ΔCMV-Firefly Luciferase as control. | 1. RNR and μDys treated mdx mice showed improved survival rate, however, not statistical significant.  2. RNR treatment normalized diastolic response, while both RNR and μDys treatments improved systolic function. | RNR therapy shows potential as a supplemental therapy, in combination with μDys treatment, however, future studies are required. |
| Xu, *et al*., 2019 [80] | To investigate whether rAAVrh74MCK.GALGT2 gene therapy can prevent loss of heart function in *mdx* mouse. | rAAVrh74.MCK.GALGT2 | Gene transfer | A study to assess the potential of GALGT2 in preventing loss of cardiac function in mdx mice, which will be performed via AAV-mediated gene therapy. | 1. Intravenous treatment significantly improved cardiac output and stroke volume in *mdx* mice.  2. Utrophin expression in *mdx* heart as a result of GALGT2 overexpression. | Overexpression of GALGT2 demonstrated inhibition of loss of cardiac function, which has therapeutic potential in DMD patients. |
| Chicoine, *et al*., 2014b [81] | To investigate whether GALGT2 can induce dystrophin and laminin α2 surrogate expression | rAAVrh74.MCK.GALGT2 | Gene transfer | A study employing AAV-mediated *in vivo* intramuscular and focal limb perfusion delivery of GALGT2 into tibialis anterior muscles of Rhesus macaques, and assessing its potential in inducing the expression of dystrophin and laminin α2 surrogates. | 1. GALGT2 was expressed in all myofibers following intramuscular delivery, however, expression rate in vascular delivery is influenced by preexisting antibodies towards the vector.  2. Overexpression of GALGT2 resulted in expression of laminin α2 surrogates. | GALGT2 overexpression had induced the expression of the proteins of interest, however, there were no significant changes to the DMD phenotype in treated macaques. The issue on possibilities of triggering an immune response, long-term, in humans was addressed considering that animal models may not reflect the full extent of AAV mediated gene transfer. |
| Heller, *et al*., 2013 [82] | To study whether upregulation of α7 integrin can improve dystrophic phenotypes in. | rAAV8.MCK.ITGA7 | Gene transfer | A study employing AAV-mediated gene transfer for in vivo delivery of ITGA7 in mdx mouse via isolated limb perfusion. | 1. Improved dystrophic pathologies, and muscle membrane integrity in terms of sarcolemmal integrity and muscle fiber diameter.  2. α7 integrin overexpression protects against eccentric contraction-induced injury. | Vector provides safe and effective delivery of ITGA7 resulting in protection against induced injury, showing promise as a feasible therapeutic approach. |
| Hakim, *et al*., 2017 [83] | To evaluate potential of mini-dystrophin genes in disease rescue by improving dystrophic phenotypes in severe DBA/2J-mdx mouse model. | Five-repeat micro-dystrophin gene | Micro-dystroph-in gene therapy | A study employing AAV-9-mediated delivery of micro-dystrophin constructs containing spectrin-like repeats, and its ability to ameliorate dystrophic phenotype of the severe DBA/2J-mdx mouse model. | 1. Micro-dystrophin was expressed body-wide with successful sarcolemmal localization and restored expression of dystrophin-associated glycoprotein complex.  2. Improved DMD muscle pathology and muscle function.  3. Effect on cardiac function, however, is not statistically significant. | Substantial improvements of muscle pathology following robust dystrophin rescue highlights the potential of the construct, however, limitations were also revealed prompting further development of the five-repeat micro-dystrophin gene. |
| Mendell, *et al*., 2020 [84] | To identify safety and tolerability of intravenous rAAVrh74.MHCK7.micro-dystrophin in patients with DMD | rAAVrh74.MHCK7.micro-dystrophin | Micro-dystroph-in gene therapy | An phase 1/2a, open label nonrandomized controlled trial with 3-year follow up on 4 patients | Robust transgene expression in gastrocnemius muscle biopsies | Vector well tolerated with minimal adverse effects |
| Bostick, *et al*., 2012 [85] | To study whether AAV-mediated micro-dystrophin gene therapy can alleviate DMD heart disease. | Micro-dystrophin gene AAV-9 vector | Micro-dystroph-in gene therapy | A novel study on the effects of *in vivo* micro-dystrophin gene therapy in end-stage model of DMD cardiomyopathy in mdx mice. | 1. Micro-dystrophin robustly expressed in the myocardium of treated mice.  2. However, no significant reduction in myocardial fibrosis.  3. Overall heart performance did not improve post-AAV microgene treatment.  4. Treated mice was more resistant to dobutamine-induced cardiac death. | While no significant improvements were shown in treated mice, the study highlighted potential benefits, and most importantly the limitations of such approach in severe Duchenne cardiomyopathy in end-stage DMD. |
| Chicoine, *et al*., 2014a [86] | To study the efficacy of micro-dystrophin gene therapy post elimination of AAV binding antibodies via plasmapheresis. | rAAVrh.74.MCK.micro-dys.FLAG | Micro-dystroph-in gene therapy | A study employing plasmapheresis in removal of AAVrh.74-binding antibodies to improve *in vivo* micro-dystrophin expression in rhesus macaques. | 1. Effective expression of the vector-mediated micro-dystrophin delivery in gastrocnemius of treated rhesus macaques.  2. Plasmapheresis reduced AAV-binding antibodies up to 10-folds.  3. Removal of AAV-binding antibodies improved gene expression by 4-folds. | The study provides implications to possible therapeutic approaches considering removal of preexisting vector antibodies allowed sustained gene transfer. |
| Hayashita-Kinoh, *et al*., 2015 [87] | To examine the therapeutic potential of rAAV-mediated microdystrophin gene transfer in canine X-linked muscular dystrophy (CXMD). | rAAV-CMV-microdystrophin | Microdystrophin gene transfer | A study employing intra-amniotic administration of rAAV-mediated microdystrophin gene in beagle-based CXMD model | 1. Transduction with intra-amniotic vector showed prominent marker gene expression. Additional systemic rAAV-microdystrophin injection showed improved marker gene expression and dystrophin expression.  2. Improved gait and cardiac function in rAAV-microdystrophin treated dog. | Long-term transgene expression was achieved, supporting the therapeutic benefits and feasibility of rAAV-mediated therapeutic approaches. |
| RNA interference | | | | | | |
| Fukuoka, *et al*., 2021) [88] | To investigate the association between cf-MiRNA, miR-199-3p and aging. | miR-199-3p | RNA interference | A study on the use of miR-199-3p in inducing myogenic differentiation in C1C12 cells, and in vivo in C57BL/6J male mice. | 1. miR-199-3p participates in myogenic differentiation.  2. miR199#4 (miR-199 mimic) significantly enhanced muscle regeneration and muscle strength. | Anti-aging effect of miR-199-3p presents a potential therapeutic approach to DMD |
