# Supplementary material for: A Decade of Progress in Gene Targeted Therapeutic Strategies in Duchenne Muscular Dystrophy: A Systematic Review
Source: Front Bioeng Biotechnol. 2022 Mar 23;10:833833. doi: 10.3389/fbioe.2022.833833 (PMC8984139; doi:10.3389/fbioe.2022.833833)
Supplement: Supplementary file 4 [file Table4.DOCX]

| Risk bias domain | | Bowles ey al., 2012 | Mendell, et al., 2020 | Akpulat et al., 2018 | Blain et al., 2018 | Chen et al., 2021 | Domenger et al., 2018 | Duchene et al., 2018 | Echigoya et al., 2018 | Fukuoka et al., 2021 | Gee et al., 2020 | Goemans et al., 2016. | Jirka et al., 2017 | Koo et al., 2018 | Lattanzi et al., 2017 | Lee et al., 2018 | Lee et al., 2017 | Lu-Nguyen et al., 2017 | Lopez et al., 2020 | Pires et al., 2016 |
| --- | --- | --- | --- | --- | --- | --- | --- | --- | --- | --- | --- | --- | --- | --- | --- | --- | --- | --- | --- | --- |
| ++ | Definitely low risk of bias |  |  |  |  |  |  |  |  |  |  |  |  |  |  |  |  |  |  |  |
| + | Probably low risk of bias |  |  |  |  |  |  |  |  |  |  |  |  |  |  |  |  |  |  |  |
| -/NR | Probably high risk of bias |  |  |  |  |  |  |  |  |  |  |  |  |  |  |  |  |  |  |  |
| -- | Definitely high risk of bias |  |  |  |  |  |  |  |  |  |  |  |  |  |  |  |  |  |  |  |
| N/A | Not applicable |  |  |  |  |  |  |  |  |  |  |  |  |  |  |  |  |  |  |  |
| Clear hypothesis/ objectives | | ++ | ++ | ++ | ++ | ++ | ++ | ++ | ++ | ++ | ++ | ++ | ++ | ++ | ++ | ++ | ++ | ++ | ++ | ++ |
| Interventions clearly described | | ++ | ++ | ++ | ++ | ++ | ++ | ++ | ++ | ++ | ++ | ++ | ++ | ++ | ++ | ++ | ++ | ++ | ++ | ++ |
| Was the administered dose or exposure level adequately randomized | | ++ | N/A | N/A | N/A | N/A | N/A | N/A | N/A | N/A | N/A | N/A | N/A | N/A | N/A | N/A | ++ | ++ | N/A | N/A |
| Was allocation to study groups adequately concealed? | | N/A | N/A | N/A | N/A | N/A | N/A | N/A | N/A | N/A | N/A | N/A | N/A | N/A | N/A | N/A | N/A | N/A | N/A | N/A |
| Were experimental conditions identical across study groups | | ++ | ++ | ++ | ++ | ++ | ++ | ++ | ++ | ++ | ++ | ++ | ++ | ++ | ++ | ++ | ++ | ++ | ++ | ++ |
| Can we be confident in the exposure characterization | | ++ | ++ | ++ | ++ | ++ | ++ | ++ | ++ | ++ | ++ | ++ | ++ | ++ | ++ | ++ | ++ | ++ | ++ | ++ |
| Were the research personnel and human subjects blinded to the study group during the study | | ++ | N/A | N/A | N/A | N/A | N/A | N/A | N/A | N/A | N/A | N/A | N/A | ++ | N/A | N/A | N/A | N/A | ++ | N/A |
| Were the outcome data complete without attrition or exclusion from analysis | | ++ | ++ | ++ | ++ | ++ | ++ | ++ | ++ | ++ | ++ | ++ | ++ | ++ | ++ | ++ | ++ | ++ | ++ | ++ |
| Can we be confident in the outcome assessment | | ++ | ++ | ++ | ++ | ++ | ++ | ++ | ++ | ++ | ++ | ++ | ++ | ++ | ++ | ++ | ++ | ++ | ++ | ++ |
| Were all measured outcomes reported | | ++ | ++ | ++ | ++ | ++ | ++ | ++ | ++ | ++ | ++ | ++ | ++ | ++ | ++ | ++ | ++ | ++ | ++ | ++ |
| Were n, N and statistical methods appropriate | | ++ | ++ | ++ | ++ | ++ | ++ | + | ++ | ++ | ++ | ++ | ++ | ++ | N/A | N/A | ++ | ++ | ++ | + |
| Were adverse events reported | | ++ | ++ | ++ | ++ | ++ | ++ | ++ | ++ | ++ | ++ | ++ | ++ | ++ | ++ | ++ | ++ | ++ | ++ | ++ |

| Risk bias domain | | Sengupta, Loro, and Khurana, 2020 | Van Putten et al., 2019 | Vila et al., 2019 | Wang et al., 2018a | Wang et al., 2018b | Wang et al., 2019 | Watanabe et al., 2018 | Xu et al., 2019 | Yang et al., 2013 | Betts et al., 2012 | Bish et al., 2012 | Bostick et al., 2012 | Cazella et al., 2012 | Chicoine et al., 2014a | Chicoine et al., 2014b | Hakim et al., 2017 | Hayashita-Kinoh et al., 2015 | Heller et al., 2013 | Iyonmbe-Engembe et al., 2016 |
| --- | --- | --- | --- | --- | --- | --- | --- | --- | --- | --- | --- | --- | --- | --- | --- | --- | --- | --- | --- | --- |
| ++ | Definitely low risk of bias |  |  |  |  |  |  |  |  |  |  |  |  |  |  |  |  |  |  |  |
| + | Probably low risk of bias |  |  |  |  |  |  |  |  |  |  |  |  |  |  |  |  |  |  |  |
| -/NR | Probably high risk of bias |  |  |  |  |  |  |  |  |  |  |  |  |  |  |  |  |  |  |  |
| -- | Definitely high risk of bias |  |  |  |  |  |  |  |  |  |  |  |  |  |  |  |  |  |  |  |
| N/A | Not applicable |  |  |  |  |  |  |  |  |  |  |  |  |  |  |  |  |  |  |  |
| Clear hypothesis/ objectives | | ++ | ++ | ++ | ++ | ++ | ++ | ++ | ++ | ++ | ++ | ++ | ++ | ++ | ++ | ++ | ++ | ++ | ++ | ++ |
| Interventions clearly described | | ++ | ++ | ++ | ++ | ++ | ++ | ++ | ++ | ++ | ++ | ++ | ++ | ++ | ++ | ++ | ++ | ++ | ++ | ++ |
| Was the administered dose or exposure level adequately randomized | | N/A | ++ | ++ | N/A | N/A | N/A | N/A | N/A | N/A | N/A | N/A | N/A | N/A | N/A | N/A | N/A | ++ | N/A | N/A |
| Was allocation to study groups adequately concealed? | | N/A | N/A | N/A | N/A | N/A | N/A | N/A | N/A | N/A | N/A | N/A | N/A | N/A | N/A | N/A | N/A | N/A | N/A | N/A |
| Were experimental conditions identical across study groups | | ++ | ++ | ++ | ++ | ++ | ++ | ++ | ++ | ++ | ++ | ++ | ++ | ++ | ++ | ++ | ++ | ++ | ++ | ++ |
| Can we be confident in the exposure characterization | | ++ | ++ | ++ | ++ | ++ | ++ | ++ | ++ | ++ | ++ | ++ | ++ | ++ | ++ | ++ | ++ | ++ | ++ | ++ |
| Were the research personnel and human subjects blinded to the study group during the study | | N/A | ++ | ++ | N/A | N/A | N/A | N/A | N/A | N/A | N/A | N/A | N/A | N/A | N/A | N/A | N/A | N/A | N/A | N/A |
| Were the outcome data complete without attrition or exclusion from analysis | | ++ | ++ | ++ | ++ | ++ | ++ | ++ | ++ | ++ | ++ | ++ | ++ | ++ | ++ | ++ | ++ | ++ | ++ | ++ |
| Can we be confident in the outcome assessment | | ++ | ++ | ++ | ++ | ++ | ++ | ++ | ++ | ++ | ++ | + | ++ | ++ | + | + | ++ | + | ++ | ++ |
| Were all measured outcomes reported | | ++ | ++ | ++ | ++ | ++ | ++ | ++ | ++ | ++ | ++ | ++ | ++ | ++ | ++ | ++ | ++ | ++ | ++ | ++ |
| Were n, N and statistical methods appropriate | | ++ | ++ | ++ | ++ | ++ | ++ | ++ | ++ | ++ | ++ | + | ++ | ++ | + | + | ++ | + | + | + |
| Were adverse events reported | | ++ | ++ | ++ | ++ | ++ | ++ | ++ | ++ | ++ | ++ | ++ | ++ | ++ | ++ | ++ | ++ | ++ | ++ | ++ |

Table 1.0 (Cont.) Tabulation of risk bias analysis

| Risk bias domain | | Jirka et al., 2015 | Kolwicz et al., 2019 | Malerba et al., 2012 | Simmons et al., 2021 | Wang et al, 2013 |
| --- | --- | --- | --- | --- | --- | --- |
| ++ | Definitely low risk of bias |  |  |  |  |  |
| + | Probably low risk of bias |  |  |  |  |  |
| -/NR | Probably high risk of bias |  |  |  |  |  |
| -- | Definitely high risk of bias |  |  |  |  |  |
| N/A | Not applicable |  |  |  |  |  |
| Clear hypothesis/ objectives | | ++ | ++ | ++ | ++ | ++ |
| Interventions clearly described | | ++ | ++ | ++ | ++ | ++ |
| Was the administered dose or exposure level adequately randomized | | N/A | N/A | N/A | N/A | N/A |
| Was allocation to study groups adequately concealed? | | N/A | N/A | N/A | N/A | N/A |
| Were experimental conditions identical across study groups | | ++ | ++ | ++ | ++ | ++ |
| Can we be confident in the exposure characterization | | ++ | ++ | ++ | ++ | ++ |
| Were the research personnel and human subjects blinded to the study group during the study | | N/A | N/A | N/A | N/A | N/A |
| Were the outcome data complete without attrition or exclusion from analysis | | ++ | ++ | ++ | ++ | ++ |
| Can we be confident in the outcome assessment | | ++ | ++ | ++ | ++ | ++ |
| Were all measured outcomes reported | | ++ | ++ | ++ | ++ | ++ |
| Were n, N and statistical methods appropriate | | ++ | ++ | ++ | ++ | + |
| Were adverse events reported | | ++ | ++ | ++ | ++ | ++ |
